# Prescriber and patient-oriented behavioural interventions to improve use of malaria rapid diagnostic tests in Tanzania: facility-based cluster randomised trial

**DOI:** 10.1186/s12916-015-0346-z

**Published:** 2015-05-15

**Authors:** Bonnie Cundill, Hilda Mbakilwa, Clare IR Chandler, George Mtove, Frank Mtei, Annie Willetts, Emily Foster, Florida Muro, Rahim Mwinyishehe, Renata Mandike, Raimos Olomi, Christopher JM Whitty, Hugh Reyburn

**Affiliations:** Faculty of Epidemiology and Population Health, London School of Hygiene and Tropical Medicine, Keppel St, London, WCIE 7HT UK; Joint Malaria Programme, Kilimanjaro Christian Medical Centre, Box 2228, Moshi, Tanzania; Faculty of Public Health and Policy, London School of Hygiene and Tropical Medicine, Keppel St, London, WCIE 7HT UK; National Institute for Medical Research, Amani Centre, Tanga, Tanzania; Wellsense International Public Health Consultants, P.O. Box 788, Kilifi, Kenya; National Malaria Control Programme, Ministry of Health and Social Welfare, Ocean Road, Dar es Salaam, Tanzania; Faculty of Infectious and Tropical Diseases, London School of Hygiene and Tropical Medicine, Keppel St, London, WCIE 7HT UK

**Keywords:** Malaria, Cluster-randomised, Rapid diagnostic test, ACT, Behavioural interventions, Primary care, Tanzania

## Abstract

**Background:**

The increasing investment in malaria rapid diagnostic tests (RDTs) to differentiate malarial and non-malarial fevers, and an awareness of the need to improve case management of non-malarial fever, indicates an urgent need for high quality evidence on how best to improve prescribers’ practices.

**Methods:**

A three-arm stratified cluster-randomised trial was conducted in 36 primary healthcare facilities from September 2010 to March 2012 within two rural districts in northeast Tanzania where malaria transmission has been declining. Interventions were guided by formative mixed-methods research and were introduced in phases. Prescribing staff from all facilities received standard Ministry of Health RDT training. Prescribers from facilities in the health worker (HW) and health worker-patient (HWP) arms further participated in small interactive peer-group training sessions with the HWP additionally receiving clinic posters and patient leaflets. Performance feedback and motivational mobile-phone text messaging (SMS) were added to the HW and HWP arms in later phases. The primary outcome was the proportion of patients with a non-severe, non-malarial illness incorrectly prescribed a (recommended) antimalarial. Secondary outcomes investigated RDT uptake, adherence to results, and antibiotic prescribing.

**Results:**

Standard RDT training reduced pre-trial levels of antimalarial prescribing, which was sustained throughout the trial. Both interventions significantly lowered incorrect prescribing of recommended antimalarials from 8% (749/8,942) in the standard training arm to 2% (250/10,118) in the HW arm (adjusted RD (aRD) 4%; 95% confidence interval (CI) 1% to 6%; *P* = 0.008) and 2% (184/10,163) in the HWP arm (aRD 4%; 95% CI 1% to 6%; *P* = 0.005). Small group training and SMS were incrementally effective. There was also a significant reduction in the prescribing of antimalarials to RDT-negatives but no effect on RDT-positives receiving an ACT. Antibiotic prescribing was significantly lower in the HWP arm but had increased in all arms compared with pre-trial levels.

**Conclusions:**

Small group training with SMS was associated with an incremental and sustained improvement in prescriber adherence to RDT results and reducing over-prescribing of antimalarials to close to zero. These interventions may become increasingly important to cope with the wider range of diagnostic and treatment options for patients with acute febrile illness in Africa.

**Trial registration:**

ClinicalTrials.gov (#NCT01292707) 29 January 2011.

**Electronic supplementary material:**

The online version of this article (doi:10.1186/s12916-015-0346-z) contains supplementary material, which is available to authorized users.

## Background

Acute febrile illness is the commonest presentation in Africa, and overdiagnosis of malaria in febrile patients in Africa and Asia is a major public health problem [1-3].Over the last decade the introduction of the relatively expensive artemisinin-based combination therapies (ACT) and the subsequent appearance of artemisinin resistance in south-east Asia have driven the need to rationalise the overuse of antimalarial drugs in Africa and Asia [4]. In addition the recent decline in malaria transmission in many areas of Africa has led to increasing awareness that ‘presumptive treatment for malaria’ is often associated with neglect of non-malarial causes of fever [5,6]. The availability of affordable and reliable rapid diagnostic tests for malaria (RDTs), comparable in price and often more accurate than a standard malaria blood slide, has provided a potentially important tool to address these problems. In 2010 the ‘WHO Guidelines for Malaria Diagnosis and Treatment’ replaced the policy of presumptive treatment for malaria in children with no obvious alternative cause of fever with a policy of recommending parasitological confirmation in all patients with suspected malaria before treatment wherever possible and restricting antimalarial treatment to parasite-positive patients [7,8].

Providing new tools does not however necessarily change practice. A large scale-up in the deployment of malaria RDTs by national malaria control programmes from less than 200,000 in 2005 to more than 108 million in 2012 has been undertaken [9]. However, increased use of parasitological tests of malaria to guide treatment often fails to achieve its objective due to lack of infrastructural and social support for denying antimalarial drugs, leading to a persistent preference among prescribers for a diagnosis of malaria, even in the face of a negative test result. In 1997 increased availability of blood slide microscopy in Zambia had little effect due to the tendency to prescribe antimalarial treatment on clinical grounds (‘presumptive treatment’) or to slide-negative patients and almost a decade later a Tanzanian study found that half of test-negative outpatients provided with RDTs and basic training were prescribed an antimalarial drug and this did not vary whether tested by RDT or blood slide [10,11]. A number of more recent studies have shown large variability in adherence to current guidelines for malaria diagnosis in both Africa and Asia, but the overall problem of preference for a diagnosis of malaria has generally prevailed [12-16]. This reduces both the clinical effectiveness and cost-effectiveness of RDTs [17], and leads to persistent overdiagnosis of malaria. Getting misdiagnosis of febrile illness as malaria close to zero should lead to improved case management and allow routine data to be used for public health. It will also be essential in areas where local elimination is the aim.

The increasing investment in RDTs and awareness of the need to improve case management of non-malarial fever indicate an urgent need for high quality evidence on how best to improve prescribers’ use of RDTs and adherence to the results. The reasons for overprescription of antimalarials even when test results are negative are complex, and depend on prescriber perceptions including of patient expectations [18]. We therefore hypothesised that interventions aimed at patient perceptions would reinforce those targeting prescribers. However, despite the large number of training interventions with prescribers in Africa, there is no strong evidence to guide the most effective format, content and techniques to change prescribing practices [19]. Few, if any, interventions have been tested that explicitly aim to intervene on the perception of prescribers of what patients want in terms of prescription in these settings. We thus conducted the Targeting Artemisinin Combination Trial (TACT), a stratified cluster-randomised trial in primary care facilities in north east Tanzania of prescriber and patient-oriented behavioural interventions to improve adherence to national and WHO malaria diagnosis and treatment guidelines. The design of interventions was guided by formative mixed-methods research, to understand the existing scenario of malaria diagnosis and antimalarial use within facilities in the trial area, and the need for simplicity and affordability if trial results are to be scaleable [19]. This formative research took an ‘evidence-based’ approach to the intervention design comprising five key stages: 1) focus group discussions and in-depth interviews with health workers and community members to understand the existing scenario of malaria diagnosis and antimalarial use; 2) a review of evidence and engagement in behaviour change theory to guide choice of intervention strategies; 3) a structured project workshop to bring together findings from previous stages into a draft outline of intervention activities and key messages; 4) designing the intervention materials; and 5) piloting and pre-testing the intervention materials.

## Methods

### Study design and participants

**Figure 1:**
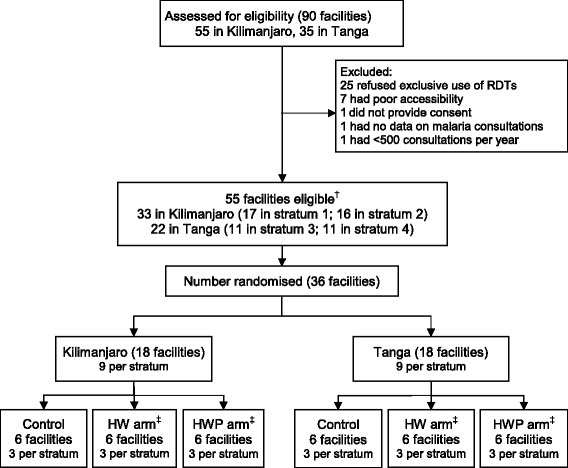
Flow of facilities through assessment of eligibility, selection and randomization. ^†^ Strata 1 and 3 had fewer malaria cases in Kilimanjaro and Tanga, respectively, while strata 2 and 4 had more malaria cases when dividing the districts into two equal categories based on the proportion of malaria consultations. Equal numbers of facilities were randomized to each arm within strata. ^‡^ Control represents the standard RDT training arm. HW represents the health worker intervention arm. HWP represents the health worker and patient-oriented intervention arm.

Between September 2010 and March 2012 we conducted a three-arm stratified cluster randomised trial among 36 facilities (clusters) within two predominately rural districts, Muheza in the Tanga region and Moshi Rural in the Kilimanjaro region, in northeast Tanzania. Malaria transmission is moderate in Muheza and low in Moshi, and has been declining over the past decade [20]. Adherence to RDT and slide results has been shown to be very poor in previous studies even after basic training [11,21]. Due to the differing transmission intensities and previous research findings we expected that the primary outcome would vary considerably across the clusters within and between the districts. We therefore stratified both by district and the proportion of all consultations that were diagnosed with malaria in the previous year, as reported in the routine Health Management Information System (MTUHA book). Within each district facilities were ranked according to the proportion of malaria consultations and split into two equal categories, giving a total of four strata (Figure 1).

Primary care facilities registered with the District Medical Office were eligible for inclusion if: they were in receipt of supplies of recommended antimalarial drugs from the Ministry of Health; qualified for RDT supply from the Government and agreed to exclusive use of RDT for routine diagnosis of suspected malaria; were accessible by four-wheel drive vehicle throughout the year; and facility data confirmed that there were more than 500 malaria diagnoses in the previous year. All prescribers employed at the facilities at any point during the trial duration were eligible to receive the intervention. On average, more than 75% of health workers at the study facilities were regular prescribers. Consenting patients who had not been referred to the next level of care were also eligible for inclusion. The nature and purpose of the trial was explained to participants and written informed consent was sought from heads of the facilities and prescribers, prior to randomisation. Participants were informed of the trial through leaflets and posters displayed in facilities’ waiting areas and health workers obtained verbal consent from patients, or carers for patients under the age of 15, to participate prior to their consultation.

### Selection of facilities, randomisation and blinding

Within each stratum, facilities were selected at random from those eligible using a computer-generated programme. Randomisation to the three trial arms was conducted within each stratum through a process of restricted (constrained) randomisation, to ensure marginal balance across the strata and study groups on covariates expected to be important correlates of the primary outcome [22]. Balance was considered achieved when: the number of health workers differed by no more than three between the study arms; number of prescribers differed by no more than two; the two mission facilities were in different arms; and the proportion of all consultations that were diagnosed with malaria in the previous year differed by less than 10%. The validity of the randomisation was assessed by examining the proportion of times triplets of clusters were allocated to the same study arm for under- and over-representation [23-25]. Selection and randomisation of facilities was conducted by the trial statistician who was not involved in the delivery of the intervention or assessment of the study outcomes using a program written in R statistical software version 2.13.0 (R Foundation for Statistical Computing, Vienna, Austria). We were not consistently able to blind patients, those delivering the interventions, or assessors of the study outcomes; however assessors were rotated through study arms every three months.

### Interventions

**Figure 2:**
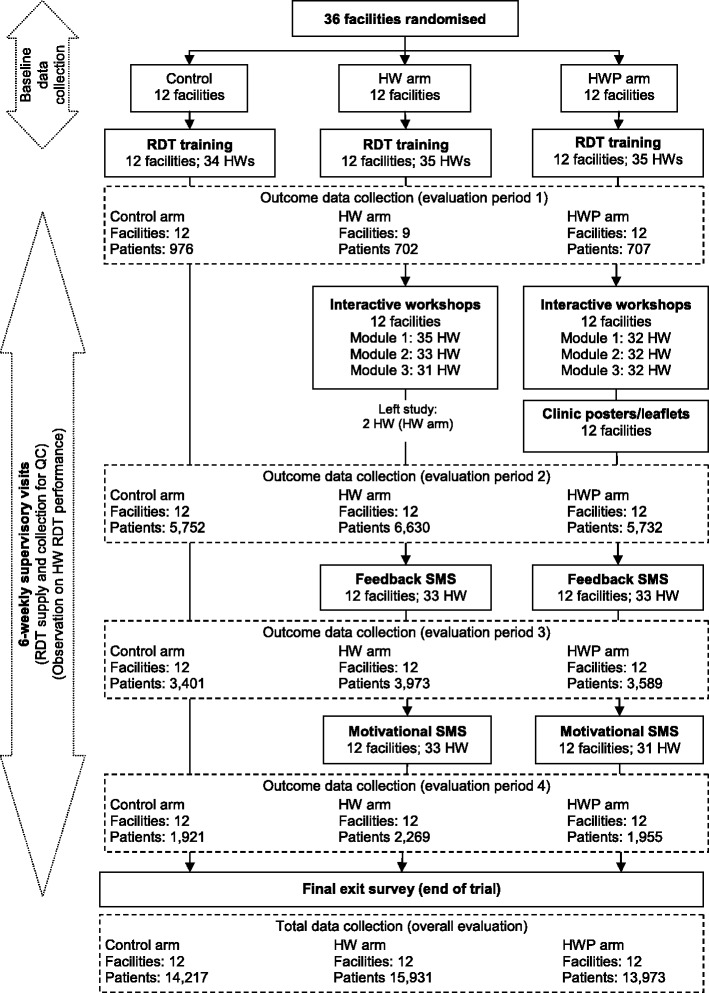
Flow of facilities, health workers (prescribers) and patients through different stages of the intervention and evaluation. The outcome data collection periods include eligible patients presenting at the facilities between the intervention implementation activities. For example, evaluation period 1 commences after the standard RDT training and initial RDT supply until the start of the intervention training. See Table 1 for further details on timing of intervention implementation and evaluation. The total data collection is based on all eligible patients presenting at the facilities following the standard RDT training until the final exit survey. It, therefore, includes patients presenting during the intervention implementation activities which were excluded in the outcome data collection periods. RDT, rapid diagnostic test.

The three arms of the trial were: 1) the standard training arm (termed control); 2) the health worker (HW) intervention arm; and 3) the health worker plus patient-oriented (HWP) intervention arm. The final multi-level intervention targeting individual prescribers at the trial facilities as well as their interaction with patients included: small group workshops, feedback and motivational mobile-phone text messages (SMS) to all prescribers in the intervention facilities and patient leaflets and clinic posters to the HWP facilities (Table 1, Figure 2). The rationale and details of the behavioural interventions in each trial arm are detailed elsewhere [19] but described in brief below.

**Table 1:** **Timelines for intervention implementation, outcome data collection and evaluation**

**Intervention component/Data collection period**	**Description of the evaluation period**	**Timescales**
Standard RDT training		18 Jan to 28 Jan 2011
RDT supply		22 Feb 2011 to 31 Jan 2012
Evaluation period 1	Commenced following standard RDT training and RDT supply until the start of the interactive workshops	4 to 6 weeks
		24 Feb to 3 April 2011
Interactive workshops		4 April to 18 May 2011
Evaluation period 2	Commenced once the interactive workshops had been completed until the start of the feedback SMS	approx 20 weeks
		19 May to 9 Oct 2011
Feedback SMS		10 Oct 2011 to 12 Mar 2012
Evaluation period 3	Commenced when the feedback SMS were introduced until the start of the motivational SMS	13 weeks
		10 Oct 2011 to 8 Jan 2012
Motivational SMS		9 Jan to 12 Mar 2012
Evaluation period 4	Commenced once the motivational SMS were introduced until the final exit survey	6 to 9 weeks
		9 Jan to 12 Mar 2012
Final exit survey		22 Feb to 12 Mar 2012
Overall evaluation period	Commenced following the introduction of the RDTs until the final exit survey	22 Feb 2011 to 12 Mar 2012

RDT, rapid diagnostic test; SMS, mobile-phone text messages.

Baseline data were collected from all randomised facilities between September 2010 and January 2011. Following baseline data collection each consenting prescriber from the facilities in all arms of the trial attended the Ministry of Health’s existing two-day RDT training (approximately 30 to 50 participants), delivered by approved National Malaria Control Programme (NMCP) trainers [26]. This was followed by a visit to the facilities by research staff when RDTs and associated supplies were provided. This defined the beginning of the evaluation period of the trial in February 2011. All facilities were also visited four to six weekly by a trained research assistant who provided essential supplies including RDTs, recorded dates of any stock outs of RDTs and ACT, and observed prescribers’ performance in RDTs if they were treating patients who required testing during the visit. Prescribers use of RDTs was checked for following the test procedure as presented in the standard training. No other aspect of the consultation was documented or commented upon.

In addition to the standard RDT training prescribers from facilities randomised to the HW and HWP arms received further training through three interactive workshops four to six weeks later. These were of approximately two hours with a small group of four to eight prescribing colleagues from neighbouring facilities, led by a group moderator from the project. Workshops followed three pre-written modules based on three stages of a change process: preparing, experimenting and consolidating prescribing change. The first module aimed to sensitise prescribers to the TACT trial and the rationale for the change in policy for management of febrile illness in order that individuals and peers consider if and how to change practice. The second module aimed at providing prescribers with confidence when using RDTs, in particular the capacity to communicate effectively, including negotiating with patients who disagree with the prescribed clinical management. The final module was aimed at sustaining the change in practice by using challenging role-plays to practice integration of RDTs and demonstrate the capacity to problem solve a RDT logistical challenge. The groups were encouraged to share experiences and to work together to identify solutions.

Prescribers in the HWP arm additionally received a supply of patient leaflets and clinic posters designed to influence prescribers through encouraging demand for RDTs and adherence to results as best practice by patients, following action research with community members and several rounds of pretesting. These were to be displayed and distributed at the facilities for the duration of the trial. Patients were not asked or expected to make any particular response to the prescriber, although the prescriber could use the leaflets to explain their decision making if they wished to do so.

Data on the implementation of the training were collected through self-filled questionnaires for participants and trainers, self-reflection and feedback from trainers, observations of training modules, and in-depth interviews.

Approximately five months after the interactive workshops until the end of the trial, prescribing staff in both intervention arms were sent a series of SMS to reinforce the aims of the workshops; namely, to build prescribers’ motivation, skills and confidence to implement the strategy of RDTs in the realities of their own practice. Initially they provided a feedback summary to prescribers of their previous month’s performance on the use of RDTs (proportion of eligible patients who were tested) and treatment prescribed based on RDT results (proportion of patients with a negative test treated with an antimalarial drug). These were then followed by motivational SMS twice a day over a 15-day period with a message on malaria case management alternated with a motivational proverb [27].

Implementation and evaluation of the intervention activities lasted 13 months and the end of the trial was defined by a one-week recording of RDT and blood slides for all consenting patients exiting the trial facilities.

### Outcomes

The primary outcome was the proportion of patients with a non-severe, non-malarial illness being incorrectly prescribed a (recommended) antimalarial in a new consultation. A non-severe illness was defined as an illness not resulting in referral to the next level of care. Non-malarial was defined as a negative RDT result or no history of fever in the previous two days of a new consultation or an obvious alternate diagnosis (soft tissue, ear or urine infection). A recommended antimalarial was quinine for children under two months old, quinine or artemether-lumefantrine (ALu – the first-line ACT in Tanzania) for women of childbearing age (15 to 45 years), and ALu for all others. Secondary outcomes examined in more depth the use of RDTs and adherence to test results as well as treatment with antibiotics.

Outcomes were measured through an interviewer-administered survey administered to all eligible and consenting patients (or caretakers) exiting the trial facilities. The survey was conducted on randomly varied two days blocks per week by survey staff recruited from the nearby population using criteria of literacy and availability and given two days of training on site. All patients exiting a consultation were briefly interviewed to determine if they had suspected malaria and if so whether they had been prescribed an antimalarial or antibiotic and if they had been tested by a RDT. Prescribers were also asked to record the same information as the exit survey as part of routine Health Management Information System (MTUHA book). These records acted as a secondary source to supplement the exit survey.

Assuming that at least 30% of patients with a non-malarial illness are treated with an antimalarial in the control arm, and a coefficient of variation between facilities within stratum of 0.25, we calculated that 12 facilities per arm and 8 non-malarial patients per facility per week would give 80% power to detect an absolute reduction from 30% to 20% in the primary outcome, at the 5% significance level [28].

RDTs (Paracheck™) were subject to national quality control measures as follows: 10 tests from each 1,000 tests supplied within a single batch were be sent for testing against known histidine-rich protein II (HRP-2) solutions maintained by the Ifakara Health Research and Development Centre in Tanzania. Tests were stored at recommended temperatures (that is, above freezing and <40°C) monitored by high and low reading thermometers. At supervision visits a single test was taken from each 25-test box and tested against whole blood containing 2,000 parasites per microlitre. Test-negative RDTs against this standard were replaced by a box of new tests. At the end of the trial the RDTs and blood slides from all patients were used to compare the accuracy of RDT against research-quality slide reading. Paracheck is recommended by the NMCP in Tanzania and has been shown to reach high levels of accuracy in East Africa [29,30].

### Statistical methods

All data were double-entered using Microsoft Access 2007 (Microsoft Inc., Redmond, WA, USA) and analysed using STATA version 12.0 (STATA Corporation, College Station, TX, USA). Analysis was by intention-to-treat and took into account between-facility variations in treatment according to guidelines using methods suitable for stratified cluster randomised trials with fewer than 20 clusters per arm [22].

For each outcome, the risk difference (RD) in each intervention arm relative to the control was computed from the mean risks across facilities in each arm and stratum. An overall estimate of the RD was calculated as the weighted average of the stratum-specific RDs. The weights were proportional to the number of facilities per stratum for comparisons where there were even numbers of facilities allocated to the study arms within each stratum, or inversely proportional to the stratum-specific variances in cases with uneven number of facilities. Corresponding 95% confidence intervals (CI) were obtained and formal hypothesis testing (at the 5% significance level) was assessed by carrying out a stratified t-test on the RD. Adjustment for covariates was made by fitting a logistic regression model using data on individuals, and including terms for stratum and the covariates of interest. Expected numbers with the outcome were computed, and compared with the observed values to provide difference-residuals for each facility. The above methods for estimating the RDs, 95% CIs and hypothesis testing were calculated as before with the residuals replacing facility-specific risks.

### Ethics and trial registration

The study was approved by the Ethical Review Boards of the National Institute for Medical Research in Tanzania (NIMRlHQ/R.8cNol. 11/24) and the London School of Hygiene and Tropical Medicine (#5877). The trial was prospectively registered with ClinicalTrials.gov (Identifier # NCT01292707). An independent data safety monitoring board monitored the trial and approved the statistical analysis plan.

## Results

Of the 55 eligible facilities, 36 were selected and randomised (12 per arm) and all are included in the analysis of the primary outcomes. A total of 44,121 eligible patients, 14,217 in the control arm, 15,931 in the HW arm, and 13,973 in the HWP arm, provided consent to participate in the study from the beginning of the trial in February 2011 until the end of the trial in March 2012 and are included in the analysis (Figures 1 and 2). All eligible patients presenting at the facilities were included in the evaluation regardless of whether the health worker they saw during the consultation was a prescriber who received training as part of the trial.

### Implementation of the intervention

All facilities received the intervention package that they were randomised to. Prescribers from all facilities received the standard RDT training and the additional prescriber training in the HW and HWP arms. Each facility was represented by, on average, three prescribers (range two to five) at the standard (baseline) RDT training and three prescribers (range one to five) at each of the interactive workshop modules. Training materials were delivered as planned and well received. In the HWP arm all facilities were provided with clinic posters and patient leaflets. Observations of prescriber performance (n = 143) were conducted in all facilities, except one in the HWP arm, and showed good adherence (>90%) with RDT procedures (such as RDTs only opened immediately before use and RDT negative declared after 15 minutes) throughout the duration of the evaluation period.

At least half of the facilities, six (50%) in the control arm, seven (58%) in the HW arm and six (50%) in the HWP arm, recorded a stock-out of RDTs at any time during the trial. ACT stock-outs at least once during the study period were experienced by six (50%) facilities in the control arm, five (42%) in the HW arm, and eight (67%) in the HWP arm. The median duration of RDT stock-outs was 20 days (range 1 to 28 days) and 41 days (range 1 to 83 days) for initial ACT stock-outs.

### Characteristics of the study population

The stratification and restricted randomisation were shown to have provided comparable study arms that were generally similar in their characteristics (Tables 2 and 3), but with some exceptions in the proportion of patients presenting with fever or history of fever, and the prescribing of antimalarials and antibiotics.

**Table 2:** **Characteristics of the facilities and prescribers in the study**

**Characteristics**	**Control**	**HW arm**	**HWP arm**
**FACILITY (CLUSTER) LEVEL**			
	**N**_**c**_ **= 12** ^**a**^	**N**_**c**_ **= 12**	**N**_**c**_ **= 12**
**Number of health workers per facility (median, range)**	4 (2 to 13)	4 (2 to 11)	4 (2 to 7)
**% of health workers who are regular prescribers (median, range)**	80% (15 to 100%)	100% (20 to 100%)	75% (29 to 100%)
**Facility type**			
Government	12 (100%)	11 (92%)	11 (92%)
Mission	0 (0%)	1 (8%)	1 (8%)
**% Consultations diagnosed with malaria per year** ^**b**^ **(mean, SD)**			
<5 years of age	37% (17%)	34% (17%)	34% (20%)
≥5 years of age	32% (13%)	32% (14%)	33% (19%)
**% Consultations treated with** ^**c**^ **: (mean, SD)**			
AM	52% (16%)	48% (20%)	40% (21%)
Recommended AM^**d**^	46% (15%)	41% (20%)	31% (20%)
Antibiotics	64% (15%)	67% (11%)	62% (10%)
**% Consultations presenting with fever treated with** ^**c**^ **: (mean, SD)**			
Antimalarial (AM)	68% (20%)	67% (27%)	63% (29%)
Recommended AM^**d**^	61% (19%)	58% (26%)	50% (29%)
Antibiotics	63% (17%)	66% (13%)	58% (15%)
**% Consultations presenting without fever treated with** ^**c**^ **: (mean, SD)**			
Antimalarial (AM)	15% (12%)	15% (14%)	7% (8%)
Recommended AM^**d**^	12% (12%)	12% (13%)	4% (3%)
Antibiotics	70% (14%)	70% (16%)	69% (8%)
**Provision of malaria training materials**			
Posters	8 (67%)	9 (75%)	7 (58%)
Books	9 (75%)	10 (83%)	9 (75%)
**MoH monitoring visit in the past year**	12 (100%)	12 (100%)	11 (92%)
**PRESCRIBER LEVEL**			
	**N**_**hw**_ **= 35** ^**a**^	**N**_**hw**_ **= 35**	**N**_**hw**_ **= 35**
**Number per facility (median, range)**	3 (2 to 4)	3 (2 to 5)	3 (2 to 4)
**Age (years)** ^e^			
21 to 34	3 (9%)	7 (21%)	6 (17%)
35 to 44	8 (23%)	4 (15%)	7 (20%)
45 to 54	11 (31%)	18 (53%)	16 (46%)
≥55	13 (37%)	4 (12%)	6 (17%)
**Gender**			
Male	8 (23%)	10 (29%)	10 (29%)
Female	27 (77%)	25 (71%)	25 (71%)
**Highest education level**			
Primary	17 (49%)	13 (37%)	11 (31%)
Secondary	8 (23%)	13 (37%)	12 (34%)
Higher (college, training etc.)	10 (29%)	9 (26%)	12 (34%)
**Cadre** ^e^			
Clinician	12 (34%)	10 (29%)	12 (34%)
Registered nurse	7 (20%)	9 (26%)	8 (23%)
Nursing/medical attendant	16 (46%)	15 (44%)	15 (43%)
**Length of time at facility** ^**e**^			
1 to 5 years	14 (45%)	15 (48%)	16 (47%)
5 to 10 years	6 (19%)	4 (13%)	3 (9%)
More than 10 years	11 (35%)	12 (39%)	15 (48%)
**Training in past 3 years** ^**f**^			
Integrated logistics system (ILS)	10 (29%)	12 (34%)	9 (26%)
IMCI	9 (27%)	10 (32%)	13 (41%)
Malaria (not specific)	3 (9%)	4 (11%)	2 (6%)

^a^N_c_ represents the number of clusters (facilities); N_hw_ represents the number of prescribers present at the facilities who consented to participate in the study; any new prescribers throughout the study duration who consented to participate were also included. ^b^Mean (SD: standard deviation) proportion of consultations diagnosed with malaria. Based on information available 2007 to 2009. ^**c**^New consultation of a non-severe illness. Fever defined as history of fever in the past two days. ^**d**^Recommended AM defined as Quinine for children weighing less than 5 kg (assuming all children over two months of age will weigh >5 kg), ALu or quinine for women of childbearing age (age 15 to 45 years inclusive), ALu for all others. ^e^Age and cadre is missing for one prescriber in the HW arm. Time at facility missing for four prescribers in the control and HW arms and one prescriber in the HWP arm. ^f^ILS is a mobile health alert and reporting system designed to increase the visibility of logistics data and improve product availability. IMCI = integrated management of childhood illness. IMCI training missing for two prescribers in the control arm, four in HW arm, and three in HWP arm. Numbers and percentages are presented unless stated otherwise. Percentages may not add to 100 due to rounding.

**Table 3:** **Characteristics of patients included in the evaluation, by arm**

**Characteristics**	**Control**	**HW arm**	**HWP arm**
	**N**_**p**_ **= 14,217** ^**a**^	**N**_**p**_ **= 15,931**	**N**_**p**_ **= 13,973**
**Number per facility (median, range)**	1227 (477 to 2112)	1325 (295 to 2275)	1233 (560 to 1825)
**Age (years)**			
<5	5290 (37%)	6144 (39%)	4671 (33%)
5 to 15	3053 (21%)	3320 (21%)	3149 (22%)
>15	5874 (41%)	6467 (41%)	6153 (44%)
**Gender**			
Male	6308 (44%)	6810 (43%)	6128 (44%)
Female	7909 (56%)	9121 (57%)	7845 (56%)
**Presented with fever**			
No	4876 (34%)	6088 (38%)	6000 (43%)
Yes	9301 (66%)	9829 (62%)	7967 (57%)
**Ear/Soft tissue infection**			
No	12324 (87%)	14228 (89%)	12530 (90%)
Yes	1883 (13%)	1690 (11%)	1436 (10%)
**Wealth index** ^**b**^			
Poorest	117 (28%)	149 (30%)	216 (42%)
Less poor	159 (37%)	167 (33%)	152 (30%)
Least poor	150 (35%)	187 (37%)	142 (28%)

^a^N_p_ represents the number of eligible patients included in the evaluation, defined as the period between the end of the RDT training and the end of the trial. Eligible patients were those with a non-severe first consultation. ^b^Measured only in a sample of patients followed up at home 14 days after they had visited the study facility. Generated through principle component analysis (PCA) and based on ownership of household possessions (for example, electricity, radio, mobile phone, bicycle, and car), access to utilities (for example, toilet type and source of drinking water), and housing characteristics (for example, floor type, fuel) in line with DHS Wealth Index [42] and Vyas *et al*. use of PCA for socio-economic status [43]. Numbers and percentages are presented unless stated otherwise. RDT, rapid diagnostic test.

### Impact on treatment of patients with non-malarial illness

**Figure 3:**
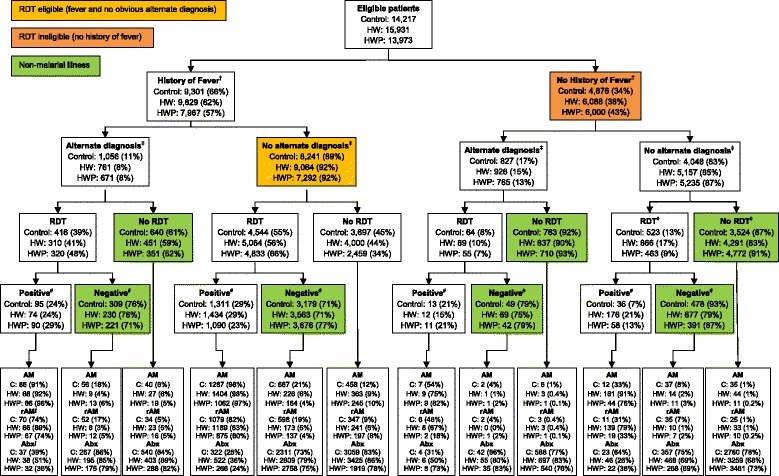
Flow chart defining the primary outcome and showing prescribing practices. ^†^ Fever status not known for 40 patients in the control arm, 14 in the HW arm and 6 in the HWP arm. Of these patients, five (13%) in the control, four (29%) in the HW arm and three (50%) in the HWP arm also had alternate diagnosis missing. Data on whether or not they had an RDT and the result is known for all patients with missing fever status but are not included in the analysis. ^‡^ Obvious alternate diagnosis (soft tissue, ear or urine infection) not known for four patients in each arm among those with a history of fever, and one in the control arm and five in the HW arm for those with no history of fever. Data on whether or not they had an RDT (and the result) is known for all these patients but are not included in the analysis. ^⋄^ Whether or not an RDT was taken is unknown for one patient in the control arm. ^#^ RDT result is unknown for 77 (1%) in the control arm, 94 (1%) in the HW arm and 92 (2%) in the HWP arm. ^∫^ Recommended antimalarial (rAM) defined as quinine for children under 2 months, Artemether Lumefantrine (ALu) or quinine for women of childbearing age, and ALu for all others. Abx represents antibiotics; HW, health worker; HWP, health worker plus patient-oriented; RDT, rapid diagnostic test.

A breakdown of the observed treatment of patients with a non-malarial illness is presented in Figure 3. Just under one-third of eligible patients attending facilities in the control (8,942/14,217 (63%)) and HW arm (10,118/15,931 (63%)), and three-quarters in the HWP arm (10,163/13,973 (73%)) were non-malarial. Of these, the percentage being incorrectly prescribed a recommended antimalarial in a new consultation was 8% in the control, 2% in the HW arm, and 2% in the HWP arm. Compared with standard RDT training, there was strong evidence that both interventions significantly lowered the prescribing of a recommended antimalarial, even after adjusting for study design and differences observed at baseline (Table 4). The adjusted risk difference (aRD) showed an absolute 4% (95% CI 1% to 6%; *P* = 0.008) reduction for the intervention focusing on prescribers only (HW arm), and a 4% reduction (95% CI 1% to 6%; *P* = 0.005) for the intervention focusing on both the prescribers and patients (HWP arm). Similar results were observed for patients <5 years and ≥5 years (Table 4), and when the analysis was restricted to those patients who 1) visited facilities when there was no RDT or ACT stock-outs and 2) had a consultation with a prescribing health worker who had attended the intervention training workshops (Additional files 1 and 2).

**Table 4:** **Effect of interventions on recommended antimalarial prescribing among patients with a non-severe, non-malarial illness**

**Evaluation category**	**Arm**	**Number of clusters**	**Number of patients**	**Prevalence number (%)**	**Crude RD** ^**a**^ **(95% CI)**	**Adjusted RD** ^**b**^ **(95% CI)**	***P*** **-value**
Overall^c^(all ages)	Control	12	8942	749 (8%)	0	0	
	HW	12	10118	250 (2%)	0.06 (0.04, 0.09)	0.04 (0.01, 0.06)	0.008
	HWP	12	10163	184 (2%)	0.07 (0.04, 0.09)	0.04 (0.01, 0.06)	0.005
<5 years	Control	12	3139	392 (12%)	0	0	
	HW	12	3682	153 (4%)	0.09 (0.04, 0.14)	0.06 (0.006, 0.12)	0.03
	HWP	12	3406	95 (3%)	0.09 (0.03, 0.14)	0.05 (0.005, 0.09)	0.03
≥5 years	Control	12	5803	357 (6%)	0	0	
	HW	12	6436	97 (2%)	0.05 (0.03, 0.07)	0.02 (0.006, 0.04)	0.008
	HWP	12	6757	89 (1%)	0.05 (0.03, 0.07)	0.03 (0.01, 0.05)	0.002
**By Evaluation period**							
Standard training - period 1 (all arms)	Control	12	656	48 (7%)	0	0	
	HW	9	449	3 (1%)	-	-	-
	HWP	11	494	38 (8%)	0.001 (−0.09, 0.09)	−0.01 (−0.06, 0.03)	0.54
Interactive training - period 2 (HW and HWP arms)	Control	12	3320	236 (7%)	0	0	
	HW	12	3791	135 (4%)	0.05 (0.02, 0.08)	0.02 (−0.001, 0.05)	0.06
	HWP	12	3802	81 (2%)	0.05 (0.02, 0.09)	0.03 (0.003, 0.05)	0.03
Feedback SMS – period 3 (HW and HWP arms)	Control	12	2392	215 (9%)	0	0	
	HW	12	2829	58 (2%)	0.06 (0.04, 0.09)	0.02 (−0.001, 0.05)	0.06
	HWP	12	2891	24 (1%)	0.07 (0.04, 0.10)	0.02 (−0.004, 0.04)	0.09
Feedback + proverb SMS - period 4 (HW and HWP arms)	Control	12	1297	106 (8%)	0	0	
	HW	12	1540	18 (1%)	0.07 (0.05, 0.09)	0.03 (0.007, 0.06)	0.01
	HWP	12	1607	7 (0.4%)	0.07 (0.05, 0.09)	0.03 (0.01, 0.06)	0.009

^a^Adjusted for stratification, effect estimate is risk difference = control– intervention; control is standard RDT training. ^b^Adjusted for facility (stratum, stock-out of ACT, provision of materials, % fever consultations treated with antimalarial prior to the study, % non-febrile consultations treated with antimalarial prior to the study), prescriber (age, education, time at facility) and patient (age) characteristics.

- Insufficient number of clusters or sample size per cluster to conduct a robust analysis. ^c^Defined as the period of evaluation from the end of the standard RDT training until the end of the trial. The between-cluster coefficient of variation was estimated as k = 0.02 for comparison of both intervention arms with the control. ACT, artemisinin-based combination therapies; CI, confidence interval; HW, health worker; HWP, health worker plus patient-oriented; RD, risk difference; RDT, rapid diagnostic test; SMS, mobile-phone text message.

Incorrect prescribing of an antimalarial for non-malarial illness was lower in the two intervention arms after the introduction of each component of the intervention package (Table 4). After the introduction of the standard RDT training, prescribing of a recommended antimalarial was low in all arms and there was a trend towards increased benefit of introducing feedback and motivating SMS to reinforce the training that had been received. There did not appear to be a waning of the effect of the standard RDT training.

### RDT uptake and adherence

There was no evidence of a significant difference in the proportion of patients presenting with a reported fever who were tested with a RDT between the trial arms (Table 5). There was, however, evidence that RDT eligible patients (presenting with fever and no obvious alternate diagnosis) were more likely to be tested in the HWP arm (66% tested) compared with standard training (55% tested); aRD 18% (95% CI 5% to 32%; *P* = 0.01). Few afebrile patients were tested with an RDT, but of those who were tested up to one-fifth (21%) were positive, including when there was an obvious alternate diagnosis (Figure 3).

**Table 5:** **Effect of interventions on antimalarial prescribing, RDT use and antibiotic prescribing**

**Outcome**	**Arm**	**Number of patients**	**Prevalence number (%)**	**Crude RD** ^**a**^ **(95% CI)**	**Adjusted RD** ^**b**^ **(95% CI)**	***P*** **-value**
Patients with fever treated with rAM	Control	9,231	2180 (24%)	0	0	
	HW	9,752	1700 (17%)	0.07 (0.004, 0.13)	0.03 (−0.04, 0.10)	0.44
	HWP	7,887	1304 (16%)	0.07 (0.01, 0.14)	0.05 (−0.002, 0.10)	0.06
Patients with no fever treated with rAM	Control	4,863	82 (2%)	0	0	
	HW	6,062	193 (3%)	−0.003 (−0.02, 0.01)	0.002 (−0.01, 0.01)	0.52
	HWP	5,984	40 (1%)	0.01 (−0.01, 0.03)	0.002 (−0.01, 0.01)	0.73
**RDT uptake**						
Patients with fever tested with RDT	Control	9,297	4960 (53%)	0	0	
	HW	9,825	5374 (55%)	−0.04 (−0.15, 0.07)	−0.04 (−0.20, 0.10)	0.57
	HWP	7,963	5153 (65%)	−0.12 (−0.21, −0.03)	−0.02 (−0.13, 0.09)	0.72
RDT eligible (fever and no obvious alternate diagnosis) not tested	Control	8,241	3697 (45%)	0	0	
	HW	9,064	4000 (44%)	0.04 (−0.07, 0.15)	0.06 (−0.11, 0.23)	0.44
	HWP	7,292	2459 (34%)	0.12 (0.04, 0.21)	0.18 (0.05, 0.32)	0.01
RDT ineligible (no fever) tested	Control	4,874	587 (12%)	0	0	
	HW	6,083	955 (16%)	−0.01 (−0.07, 0.04)	0.01 (−0.06, 0.07)	0.86
	HWP	6,000	518 (9%)	0.02 (−0.05, 0.09)	0.02 (−0.04, 0.09)	0.43
**Presumptive treatment**						
RDT eligible treated presumptively for malaria	Control	8,241	471 (6%)	0	0	
	HW	9,064	374 (4%)	0.02 (−0.01, 0.05)	0.01 (−0.02, 0.04)	0.40
	HWP	7,292	256 (4%)	0.02 (−0.003, 0.05)	0.02 (−0.004, 0.05)	0.09
RDT ineligible treated presumptively for malaria	Control	4,874	42 (1%)	0	0	
	HW	6,083	47 (1%)	0.004 (−0.001, 0.01)	0.003 (−0.001, 0.01)	0.15
	HWP	6,000	12 (0.2%)	0.007 (0.003, 0.01)	0.004 (−0.0001, 0.01)	0.05
**Adherence to RDT negative**						
RDT negative receiving AM	Control	4,015	762 (19%)	0	0	
	HW	4,539	250 (6%)	0.14 (0.08, 0.20)	0.10 (0.03, 0.17)	0.01
	HWP	4,330	189 (4%)	0.15 (0.09, 0.21)	0.10 (0.04, 0.16)	0.002
RDT negative receiving AM (among those with fever)	Control	3,488	723 (21%)	0	0	
	HW	3,793	235 (6%)	0.16 (0.08, 0.23)	0.11 (0.03, 0.19)	0.01
	HWP	3,897	177 (5%)	0.21 (0.04, 0.17)	0.12 (0.05, 0.19)	0.002
RDT negative receiving AM (among those with no fever)	Control	527	39 (7%)	0	0	
	HW	746	15 (2%)	0.05 (−0.01, 0.10)	0.03 (0.01 0.05)	0.004
	HWP	433	12 (3%)	0.04 (−0.01, 0.10)	-	-
**Adherence to RDT positive**						
RDT positive receiving rAM	Control	1,455	1166 (80%)	0	0	
	HW	1,696	1402 (83%)	−0.10 (−0.35, 0.15)	−0.13 (−0.45, 0.19)	0.39
	HWP	1,249	963 (77%)	−0.17 (−0.41, 0.06)	−0.04 (−0.25, 0.17)	0.69
RDT positive receiving rAM (among those with fever)	Control	1,406	1149 (82%)	0	0	
	HW	1,508	1255 (83%)	−0.07 (−0.31, 0.18)	−0.11 (−0.44, 0.22)	0.49
	HWP	1,180	942 (79%)	−0.14 (−0.37, 0.09)	−0.01 (−0.23, 0.21)	0.89
RDT positive receiving rAM (among those without fever)	Control	49	17 (35%)			
	HW	188	147 (78%)	-	-	
	HWP	69	21 (30%)	-	-	
**Treatment with antibiotics**						
Non-malarial illness receiving ABx	Control	8,942	6865 (77%)	0	0	
	HW	10,118	7886 (78%)	0.01 (−0.09, 0.12)	0.02 (−0.22, 0.24)	0.89
	HWP	10,163	7525 (74%)	0.01 (−0.04, 0.07)	0.14 (−0.01, 0.29)	0.06
RDT negative receiving ABx	Control	4,015	2977 (74%)	0	0	
	HW	4,539	3527 (78%)	−0.02 (−0.12, 0.09)	0.003 (−0.23, 0.24)	0.98
	HWP	4,330	3236 (75%)	−0.004 (−0.07, 0.06)	0.13 (−0.02, 0.27)	0.08
RDT eligible receiving ABx	Control	8,241	5731 (70%)	0	0	
	HW	9,064	6808 (75%)	−0.03 (−0.13, 0.07)	0.01 (−0.21, 0.25)	0.89
	HWP	7,292	4,994 (68%)	0.01 (−0.09, 0.10)	0.13 (−0.03, 0.30)	0.09
RDT ineligible receiving ABx	Control	4,863	3774 (78%)	0	0	
	HW	6,062	4531 (75%)	0.07 (−0.07, 0.20)	0.03 (−0.21, 0.28)	0.77
	HWP	5,984	4334 (72%)	0.04 (−0.02, 0.10)	0.15 (0.01, 0.30)	0.04

^a^Adjusted for stratification; effect estimate is risk difference = control – intervention; control is standard RDT training. ^b^Adjusted for facility (stock-out of ACT, stratum, provision of materials), prescriber (age, education, time at facility) and patient (age) characteristics. Treatment outcomes additionally adjusted for facility-level proportion treated with recommended antimalarial (rAM)/any antimalarial (AM) at baseline. Insufficient clusters per stratum and cluster size to conduct a robust analysis. Number of clusters is 12 per arm for all outcomes. ACT, artemisinin-based combination therapies; AM, antimalarial; CI, confidence interval; HW, health worker; HWP, health worker plus patient-oriented; rAM, recommended antimalarial; RD, risk difference; RDT, rapid diagnostic test; SMS, mobile-phone text message.

The prescriber and prescriber plus patient-oriented interventions significantly reduced the proportion of RDT negative patients receiving an antimalarial from 19% in the control to 6% in the HW arm (aRD = 10%; 95% CI 3% to 17%; *P* = 0.01) and 4% in the HWP arm (aRD = 10%; 95% CI 4% to 16%; *P* = 0.002). There was no evidence, however, of a significant increase in the proportion of RDT positive patients receiving an ACT in the intervention arms (80% in the control, 83% in the HW arm (aRD = −13%; 95% CI −45% to 19%), and 77% in HWP arm (aRD = −4%; 95% -25% to 17%)). Similar results were observed when analysis was restricted to patients attending facilities when there were no RDT and/or ACT stock-outs (Additional file 3).

Overall, the introduction of RDTs with the intervention packages resulted in an observed decrease in the proportion of consultations prescribed any antimalarial from 48% and 40% prior to the trial (Table 2) in the HW and HWP arms, respectively, to 15% and 12% at the end of the trial (Figure 3). In the control, the corresponding figures were 52% prior to the trial and 19% at the end of the trial.

### Prescribing of antibiotics

There was no evidence of a difference in the prescribing of antibiotics between the control and HW arms but there was evidence that the HWP interventions significantly reduced the proportion of patients with non-malarial illness receiving an antibiotic (aRD 0.14; 95% CI −0.01 to 0.29; *P* = 0.06). Similar results were observed among RDT negative patients and those RDT eligible and ineligible (Table 5). However, compared with prior to the trial, the prescribing of antibiotics has increased across all arms. Prior to the trial the observed proportion of consultations prescribed an antibiotics was 64% in the control arm, 67% in the HW arm and 62% in the HWP arm (Table 2). These figures had increased to 73%, 75% and 70%, respectively, at the end of the trial (Figure 3).

### Quality of RDT reporting

Agreement between known RDT results recorded by patient recall and the MTUHA register was high overall (98% agreement; kappa = 0.94) and in each trial arm. There was also excellent agreement (kappa = 0.87) between RDT results recorded in the MTUHA register and the random selection of RDTs interpreted by a member of the research team. In the final exit survey the sensitivity of the RDT results recorded in MTUHA register against the research blood slides (n = 105) was 89% (95% CI 52% to 98%) and the specificity was 95% (95% CI 88% to 98%).

## Discussion

Improving the quality of diagnosis at healthcare facilities requires both diagnostic tools and behaviour change of longstanding prescriber behaviour. Introducing RDTs for malaria with basic training has had some effect, but in multiple studies does not get close to zero overdiagnosis [11-16]. Fever is the commonest reason for patients presenting to clinics in Africa, and malaria the commonest diagnosis made, so even modest changes in overdiagnosis can have substantial impact on patient management and overuse of antimalarials. This large trial of behavioural interventions at the prescriber level led to a significant reduction in over-prescription but a patient-oriented intervention did not lead to further significant gains. The interventions led to a high level of adherence to results, and showed that with this combination of simple and repeatable behavioural interventions over-diagnosis of malaria could be reduced to close to zero in an area where the great majority of antimalarials used to be prescribed to people with no parasites. Near zero overdiagnosis brings considerable gains that will become increasingly important if resistance to ACTs spreads as infections occurring in the weeks following unnecessary treatment may be exposed to sub-therapeutic drug levels, particularly relevant to the longer acting ACT partner drugs such as piperaquine. In addition it allows improved diagnosis of other diseases and increases the reliability of routine data to be used to monitor malaria control.

Most studies of antimalarial drug prescribing in Tanzania and elsewhere have shown that prescribing antimalarial drugs even when presented with evidence of a non-malarial cause of fever, is a normalised and expected practice, reinforced by malaria-oriented infrastructure and disease control activities [31-33]. An important finding from the early phase of the trial is that the introduction of RDTs supported by the standard RDT training package in Tanzania was followed by a three to four fold reduction in antimalarial drug prescription in all trial arms. That prescribers changed their practice so quickly, and to the extent of almost eliminating use of antimalarial drugs for non-malarial cases in the intervention arms can be interpreted in the context of an increasing national drive for parasite-based malaria diagnosis, with a country-wide scale-up of RDTs that has been ongoing since 2010 [34] which could have raised awareness and readiness for change [35]. The additional benefit in the intervention arms may be attributed to the intervention’s emphasis on changing practice through a shared experience of the process of change; such a process has been described as using a ‘community of practice’ [36]. It could also be that the quality of delivery of the standard training in our trial may have been greater because trainers were incentivised by the study, which would point to the importance, noted by others, of the way interventions are delivered as much as the content [37].

The largest effect we observed was the improvement in adherence to RDT negative results in the health worker arm, with further marginal gains in the health worker-patient arm. This was a specific practice targeted for change by the interventions. However, while adherence to RDT negative results improved, prescribing of ACT to RDT positives was lower than we would have liked for improved malaria case management. This did not appear to be related to ACT stock-outs. Future interventions need to ensure that attention is focused on correct treatment of non-malarial febrile illnesses for which there are currently no routinely used point of care diagnostic tests in Africa. Our estimate of effect was based on a case definition of malaria that included a current or recent fever and yet over one fifth of patients who were tested without such a history actually had a positive RDT. While this may in part be due to persistence of HRP-2 a case could be made to treat these patients, particularly where malaria elimination is the goal.

The peer group workshops, the feedback SMS and the motivational SMS each appear to have contributed incremental improvements to the point where overuse of antimalarials was nearly eliminated after the maximum intervention was received. The small-group training was a strategy borrowed from resource-rich environments where physicians often participate in support groups to reflect on, and support change in their clinical and consultation skills. It builds on the finding that perceived peer pressure is one of the reasons for malaria misdiagnosis [32] and the observation that change in RDT use has occurred through informal group discussion and experimentation [38]. Formalising such a process of change requires skilled facilitators and success requires motivated health workers. These elements are often not present in resource poor settings, but our results do suggest that a measurable improvement is possible with only three attendances, which is achievable. As such, peer group training may provide a future model for in-service education beyond malaria case management, and may be useful to employ, for example, as medical practice moves from a scenario of simple guidelines with few diagnostic resources to a scenario where a wider range of diagnoses are considered and supported by more diagnostic resources. The sending of SMS to prescribers appears a low cost addition, as has been found elsewhere, although their effect in the absence of the initial training programmes cannot be established from these trials [27]. The intervention in which prescribers received patient leaflets and clinic posters as well as the interactive workshops is low cost to scale-up but it did require intensive development and pretesting with end users, and while only marginal improvements in prescribing beyond the workshops alone were observed we did observe continued improvements in the selection of patients for RDT testing. Further research would be required to establish the independent effects of each intervention component. For programme managers, achieving improved targeting of antimalarials will require a balance between the level of overuse of antimalarials they are willing to tolerate, and the level of investment in interventions they are able to make.

In our study, the pre-trial levels of antibiotic prescribing were already high, exceeding two-thirds of consultations, and this increased further following the introduction of RDTs, although prescribing was significantly lower in the prescriber and patient arm. A general increase in antibiotic prescribing has been found in other studies of the impact of RDTs on prescriber behaviour [13,39]. There is evidence that less than 10% of patients with suspected malaria and a negative RDT result has a positive blood culture, and the commonest indication for antibiotics appears to be the WHO-IMCI category of ‘non-severe pneumonia’, an indication for which a placebo controlled trial failed to demonstrate a benefit [40]. These considerations suggest the need to more clearly define indications for antibiotics in RDT negative patients with suspected malaria. Currently, there is a suggestion of some degree of substituting antimalarials for antibiotics.

Trials of behavioural interventions often suffer from a lack of sufficient formative qualitative research; a strength of the current study was an in-depth formative period. A novel feature of the trial was targeting prescribers and patients (who may influence prescribers) simultaneously. However, the study has a number of limitations in common with all trials of complex interventions. This was an intervention with multiple components and the study was not designed to distinguish their independent effects. In addition, we cannot exclude that our results are subject to participation bias, whereby behavioural outcomes can change due to evaluation activities in all arms of the study [41]. The observation of patient care can affect performance of health staff (the ‘Hawthorne Effect’), although prolonged observation reduces this effect, and was similar in all three arms all of whom had received training in RDTs so would be unlikely to be the cause for the significant effect seen. We also found no major differences in prescribers’ practices on days when there was an exit survey compared with days when there was none (Leurent *et al*., unpublished data). Finally, the proximity of facilities in different arms may have allowed some leakage of effect between arms, but this would tend to reduce effect sizes. The trial findings are likely to be generalisable to similar settings, the rural or semi-rural primary care facilities that serve much of sub-Saharan Africa.

## Conclusions

In a geographical area where previously in some areas over 90% of patients who were prescribed an antimalarial did not have malaria, this study has demonstrated that a combination of prescriber and patient behavioural interventions can, by incremental steps, take this down close to zero. The large reduction in antimalarial drug prescribing that occurred in both the control and intervention arms suggests that introducing RDTs with standard training at the primary care level is likely to have a significant impact on the overuse of ACT in primary care facilities in Africa. In addition, small group training with SMS messaging was associated with a significant and sustained improvement in prescriber adherence to RDT results. These interventions may become increasingly important as health services develop and clinical staff are required to use a wider range of diagnostic tests and treatment options for the commonest syndrome presenting to clinicians in Africa.

## Additional files

Additional file 1:
**Effect of interventions on recommended antimalarial prescribing among patients visiting facilities when there were no RDT or AL stock-outs.**
Additional file 2:
**Effect of interventions on recommended antimalarial prescribing among patients in consultation with prescriber who attended training.**
Additional file 3:
**Effect of interventions on secondary outcomes among patients visiting facilities during no RDT and/or AL stock-outs.**

## Abbreviations

Abx: antibiotics
ACT: artemisinin combination therapy
ALu: Artemether lumefantrine
AM: any antimalarial
aRD: adjusted risk difference
CI: confidence interval
HRP-2: histidine-rich protein II
HW: health worker intervention arm
HWP: health worker-patient-oriented intervention arm
NMCP: National Malaria Control Programme
rAM: recommended antimalarial
RD: risk difference
RDT: rapid diagnostic test
SD: standard deviation
SMS: mobile phone text messages
