# Supplementary material for: Prescriber and patient-oriented behavioural interventions to improve use of malaria rapid diagnostic tests in Tanzania: facility-based cluster randomised trial
Source: BMC Med. 2015 May 15;13:118. doi: 10.1186/s12916-015-0346-z (PMC4445498; doi:10.1186/s12916-015-0346-z)
Supplement: Additional file 1: — Effect of interventions on recommended antimalarial prescribing among patients visiting facilities when there were no RDT or AL stock-outs. [file 12916_2015_346_MOESM1_ESM.pdf]

Additional Table 1: Effect of interventions on (recommended) antimalarial prescribing among patients visiting facilities when there were no RDT or AL stock-outs

	Arm	Number of clusters	Number of patients	Prevalence n (%)	Crude RD† (95% CI)	Adjusted RD‡ (95% CI)	p-value
Overall§ (all ages)	Control	12	8350	692 (8%)	0	0	
	HW	12	9362	186 (2%)	0.06 (0.04, 0.09)	0.04 (0.01, 0.07)	0.007
	HWP	12	8402	145 (2%)	0.07 (0.04, 0.09)	0.04 (0.02, 0.06)	0.002
<5 years	Control	12	2880	363 (13%)	0	0	
	HW	12	3390	127 (4%)	0.09 (0.04, 0.14)	0.07 (0.002, 0.13)	0.04
	HWP	12	2753	77 (3%)	0.09 (0.03, 0.14)	0.04 (0.008, 0.08)	0.02
≥5 years	Control	12	5470	329 (6%)	0	0	
	HW	12	5972	59 (1%)	0.05 (0.03, 0.07)	0.02 (0.008, 0.04)	0.004
	HWP	12	5649	68 (1%)	0.05 (0.03, 0.07)	0.03 (0.01, 0.05)	0.001
**By Evaluation Period**							
Standard training (all arms)	Control	12	611	44 (7%)	0	0	
	HW	9	446	3 (1%)	-	-	-
	HWP	10	489	38 (8%)	-	-	-
Interactive training (HW and HWP arms)	Control	12	2892	220 (8%)	0	0	
	HW	12	3162	82 (3%)	0.05 (0.02, 0.09)	0.03 (0.007, 0.06)	0.01
	HWP	12	2915	42 (1%)	0.06 (0.03, 0.10)	0.03 (0.005, 0.05)	0.02
Feedback SMS (HW and HWP arms)	Control	12	2393	215 (9%)	0	0	
	HW	12	2807	58 (2%)	0.06 (0.04, 0.09)	0.03 (-0.001, 0.06)	0.06
	HWP	11	2393	24 (1%)	0.07 (0.04, 0.10)	0.04 (0.01, 0.07)	0.01
Feedback + proverb SMS (HW and HWP arms)	Control	12	1297	106 (8%)	0	0	
	HW	12	1504	18 (1%)	0.07 (0.05, 0.09)	0.03 (0.007, 0.06)	0.02
	HWP	12	1360	7 (0.5%)	0.07 (0.05, 0.09)	0.04 (0.02, 0.07)	0.003

Notes:
† Adjusted for stratification. Effect estimate is risk difference = control – intervention
‡ Adjusted for facility (stratum, provision of materials, % fever consultations treated with antimalarial prior to the study, % non-febrile consultations treated with antimalarial prior to the study), health worker (age, education, time at facility) and patient (age) characteristics.
- Insufficient number of clusters per stratum or sample size per cluster to conduct a robust analysis
§ Defined as the period of evaluation from the end of the standard RDT training until the end of the trial.
