# Supplementary material for: Prescriber and patient-oriented behavioural interventions to improve use of malaria rapid diagnostic tests in Tanzania: facility-based cluster randomised trial
Source: BMC Med. 2015 May 15;13:118. doi: 10.1186/s12916-015-0346-z (PMC4445498; doi:10.1186/s12916-015-0346-z)
Supplement: Additional file 2: — Effect of interventions on recommended antimalarial prescribing among patients in consultation with prescriber who attended training. [file 12916_2015_346_MOESM2_ESM.pdf]

Additional Table 2: Effect of interventions on (recommended) antimalarial prescribing among patients in consultation with prescriber who attended training

	Arm	Number of clusters	Number of patients	Prevalence n (%)	Crude RD† (95% CI)	Adjusted RD‡ (95% CI)	p-value
Overall§ (all ages)	Control	12	8437	738 (9%)	0	0	
	HW	12	8978	247 (3%)	0.06 (0.04, 0.09)	0.04 (0.01, 0.07)	0.007
	HWC	12	9875	176 (2%)	0.07 (0.04, 0.09)	0.04 (0.02, 0.07)	0.002
<5 years	Control	12	2979	382 (13%)	0	0	
	HW	12	3362	151 (4%)	0.09 (0.04, 0.14)	0.07 (0.008, 0.13)	0.03
	HWC	12	3334	91 (3%)	0.09 (0.03, 0.15)	0.05 (0.009, 0.09)	0.02
≥5 years	Control	12	5458	356 (7%)	0	0	
	HW	12	5616	96 (2%)	0.05 (0.03, 0.07)	0.02 (0.007, 0.04)	0.008
	HWC	12	6541	85 (1%)	0.05 (0.03, 0.07)	0.04 (0.02, 0.05)	0.001
**By Evaluation Period**							
Standard training (all arms)	Control	12	650	48 (7%)	0	0	
	HW	8	292	2 (1%)	-	-	-
	HWC	10	489	38 (8%)	-	-	-
Interactive training (HW and HWC arms)	Control	12	3143	235 (7%)	0	0	
	HW	12	3455	135 (4%)	0.05 (0.02, 0.08)	0.03 (-0.0004, 0.05)	0.05
	HWC	12	3766	81 (2%)	0.05 (0.02, 0.09)	0.03 (0.001, 0.05)	0.04
Feedback SMS (HW and HWC arms)	Control	12	2178	210 (10%)	0	0	
	HW	12	2546	57 (2%)	0.06 (0.04, 0.09)	0.03 (0.001, 0.06)	0.04
	HWC	12	2745	19 (1%)	0.07 (0.05, 0.10)	0.04 (0.01, 0.07)	0.007
Feedback + proverb SMS (HW and HWC arms)	Control	12	1199	102 (8%)	0	0	
	HW	12	1353	18 (1%)	0.07 (0.05, 0.09)	0.03 (0.006, 0.05)	0.02
	HWC	12	1507	4 (0.3%)	0.08 (0.06, 0.10)	0.05 (0.02, 0.07)	0.001

Notes:
† Adjusted for stratification. Effect estimate is risk difference = control – intervention
‡ Adjusted for facility (stratum, stock-out of ACT, provision of materials, % fever consultations treated with antimalarial prior to the study, % non-febrile consultations treated with antimalarial prior to the study), health worker (age, education, time at facility) and patient (age) characteristics.
- Insufficient number of clusters per stratum or sample size per cluster to conduct a robust analysis
§ Defined as the period of evaluation from the end of the standard RDT training until the end of the trial.
