# Supplementary material for: Prescriber and patient-oriented behavioural interventions to improve use of malaria rapid diagnostic tests in Tanzania: facility-based cluster randomised trial
Source: BMC Med. 2015 May 15;13:118. doi: 10.1186/s12916-015-0346-z (PMC4445498; doi:10.1186/s12916-015-0346-z)
Supplement: Additional file 3: — Effect of interventions on secondary outcomes among patients visiting facilities during no RDT and/or AL stock-outs. [file 12916_2015_346_MOESM3_ESM.pdf]

Additional Table 3: Effect of interventions on secondary outcomes among patients visiting facilities during no RDT and/or AL stockouts

	Arm	Number of patients	Prevalence n (%)	Crude RD[†] (95% CI)	Adjusted RD[‡] (95% CI)	p-value
Patients with fever treated with rAM[a]	Control	8643	2071 (24%)	1	1	
	HW	9191	1485 (16%)	0.07 (0.02, 0.13)	0.03 (-0.04, 0.10)	0.42
	HWC	6792	1046 (15%)	0.08 (0.02, 0.14)	0.05 (0.002, 0.10)	0.04
Patients with no fever treated with rAM[a]	Control	4656	79 (2%)	1	1	
	HW	5713	166 (3%)	-0.003 (-0.02, 0.02)	0.002 (-0.005, 0.01)	0.54
	HWC	4870	37 (1%)	0.01 (-0.01, 0.03)	0.002 (-0.01, 0.01)	0.75
**RDT uptake**						
Patients with fever tested[b]	Control	8977	4946 (55%)	1	1	
	HW	9445	5370 (57%)	-0.05 (-0.16, 0.06)	-0.05 (-0.22, 0.12)	0.54
	HWC	7650	5149 (67%)	-0.13 (-0.22, -0.04)	-0.02 (-0.13, 0.09)	0.70
*RDT eligible not tested[b]	Control	7959	3427 (43%)	1	1	
	HW	8719	3659 (42%)	0.05 (-0.06, 0.16)	0.07 (-0.10, 0.23)	0.39
	HWC	7016	2186 (31%)	0.13 (0.05, 0.22)	0.18 (0.05, 0.31)	0.01
*RDT ineligible (no fever) tested[b]	Control	4780	586 (12%)	1	1	
	HW	5841	955 (16%)	-0.02 (-0.07, 0.04)	0.004 (-0.07, 0.07)	0.91
	HWC	5900	518 (9%)	0.02 (-0.05, 0.09)	0.03 (-0.04, 0.09)	0.43
**Presumptive treatment**						
*RDT eligible treated presumptively for malaria[b]	Control	7959	308 (4%)	1	1	
	HW	8719	137 (2%)	0.02 (-0.01, 0.05)	0.02 (-0.004, 0.04)	0.11
	HWC	7016	71 (1%)	0.03 (0.001, 0.05)	0.02 (-0.001, 0.04)	0.07
*RDT ineligible treated presumptively for malaria[b]	Control	4780	36 (1%)	1	1	
	HW	5841	15 (0.3%)	0.006 (0.001, 0.01)	0.004 (-0.0003, 0.008)	0.07
	HWC	5900	6 (0.1%)	0.007 (0.003, 0.01)	0.004 (0.0004, 0.008)	0.03
**Adherence to RDT negative**						
*RDT negative receiving AM[a,b]	Control	3698	698 (19%)	1	1	
	HW	4328	233 (5%)	0.14 (0.08, 0.20)	0.09 (0.02, 0.15)	0.01
	HWC	3783	163 (4%)	0.19 (0.04, 0.15)	0.09 (0.03, 0.15)	0.006
RDT negative receiving AM[a,b] (among those with fever)	Control	3228	660 (20%)	1	1	
	HW	3641	220 (6%)	0.15 (0.08, 0.22)	0.10 (0.02, 0.18)	0.01
	HWC	3372	151 (4%)	0.17 (0.10, 0.23)	0.10 (0.03, 0.17)	0.006
RDT negative receiving AM[a,b] (among those with no fever)	Control	470	38 (8%)	1	1	
	HW	687	13 (2%)	0.05 (-0.01, 0.10)	0.03 (0.01, 0.05)	0.005
	HWC	411	12 (3%)	0.04 (-0.02, 0.10)	-	-

**Adherence to RDT positive**						
RDT positive receiving rAM[a,b]	Control	1272	1110 (88%)	1	1	
	HW	1422	1205 (85%)	-0.07 (-0.31, 0.16)	-0.11 (-0.43, 0.22)	0.49
	HWC	940	755 (80%)	-0.16 (-0.39, 0.06)	-0.03 (-0.25, 0.19)	0.77
* RDT positive receiving rAM[a,b] (among those with fever)	Control	1230	1103 (90%)	1	1	
	HW	1280	1083 (85%)	-0.04 (-0.27, 0.20)	-0.09 (-0.42, 0.25)	0.59
	HWC	884	736 (83%)	-0.13 (-0.35, 0.08)	0.001 (-0.22, 0.23)	0.99
RDT positive receiving rAM [a,b] (among those without fever)	Control	42	7 (17%)			
	HW	142	122 (86%)	-	-	
	HWC	56	19 (34%)	-	-	
**Treatment with Antibiotics**						
Non-malarial illness receiving ABx[a,b]	Control	8350	6383 (76%)	1	1	
	HW	9362	7324 (78%)	0.01 (-0.09, 0.12)	0.02 (-0.22, 0.26)	0.87
	HWC	8402	6258 (74%)	0.02 (-0.04, 0.08)	0.15 (-0.01, 0.31)	0.07
RDT negative receiving ABx [a,b]	Control	3698	2723 (74%)	1	1	
	HW	4328	3396 (78%)	-0.02 (-0.12, 0.09)	0.01 (-0.23, 0.26)	0.92
	HWC	3783	2830 (75%)	0.001 (-0.07, 0.07)	0.14 (-0.02, 0.30)	0.08
RDT eligible receiving ABx [a,b]	Control	7490	5258 (70%)	1	1	
	HW	8244	6367 (77%)	-0.04 (-0.14, 0.07)	0.01 (-0.22, 0.25)	0.90
	HWC	6059	4271 (70%)	-0.003 (-0.10, 0.09)	0.14 (-0.04, 0.31)	0.11

Notes: Number of clusters is 12 per arm for all outcomes

† Adjusted for stratification. Effect estimate is risk difference = control – intervention.

‡ Adjusted for facility (stock-out of ACT, stratum, provision of materials), health worker (age, education, time at facility) and patient (age) characteristics. Treatment outcomes additionally adjusted for facility-level proportion treated with rAM/AM at baseline.

- Insufficient clusters per stratum and cluster size to conduct a robust analysis.

a Evaluated among those who visited the facilities when there was no AL stock-outs.

b Evaluated among those who visited the facilities when there was on RDT stock-outs.
